# Multi‐omics and low‐input proteomics profiling reveals dynamic regulation driving pluripotency initiation in early mouse embryos

**DOI:** 10.1002/2211-5463.70184

**Published:** 2026-01-03

**Authors:** Wanqiong Li, Xi Xiao, Lingbin Qi, Chanyi Li, Sirui Song, Jiaying Qin, Zhigang Xue, Jinfeng Xue, Huaifang Li

**Affiliations:** ^1^ Department of Obstetrics and Gynecology, Tongji Hospital of Tongji University, Tongji University School of Medicine Shanghai China; ^2^ Department of Reproductive Center, Tongji Hospital, School of Medicine, Tongji University Shanghai China; ^3^ Stem Cell Research Center, Tongji Hospital, School of Medicine, Tongji University Shanghai China; ^4^ Reproductive Medicine Center, The First Affiliated Hospital of China Naval Military Medical University Shanghai China; ^5^ Department of Gynecology and Obstetrics, Development and Related Diseases of Women and Children Key Laboratory of Sichuan Province, Key Laboratory of Birth Defects and Related Diseases of Women and Children, Ministry of Education, West China Second Hospital, Sichuan University Chengdu China

**Keywords:** cytoskeletal remodeling, eight‐cell embryo, multiple omics, totipotency‐to‐pluripotency transition, ubiquitin‐proteasome pathway

## Abstract

Pre‐implantation of a mouse embryo is a process of transitioning from totipotency to pluripotency. However, the regulatory mechanisms underlining this transition remain poorly understood. We combined single‐cell transcriptomic analysis, epigenetic profiling, low‐input proteomics and functional validation experiments with the aim of gaining insight into the dynamic regulatory networks underlying the transition from the eight‐cell to the 16‐cell stage of mouse development. Transcriptomic analysis and H3K4me3 modification profiling, combined with functional half of the blastomeres biopsy, revealed that eight‐cell mouse embryos retained residual totipotency‐related molecular features and developmental potential. Furthermore, we identified dynamic cytoskeletal remodeling, regulated in part by the ubiquitin‐proteasome system, as a prominent molecular event during the transition from the eight‐cell to the 16‐cell stage. Collectively, our findings highlight the eight‐cell stage as a critical developmental window characterized by gradual restriction of developmental potential, and also underscore cytoskeletal remodeling as a key molecular process coinciding with the onset of pluripotency. These results provide insight into the biological processes underlying totipotent features and how these are progressively attenuated during early mouse embryogenesis.

## Graphical abstract

Mouse pre‐implantation development involves a transition from totipotency to pluripotency. Integrating transcriptomics, epigenetic profiling, low‐input proteomics and functional assays, we show that eight‐cell embryos retain residual totipotency features, whereas cytoskeletal remodeling regulated by the ubiquitin‐proteasome system drives progression toward pluripotency at the 16‐cell stage.

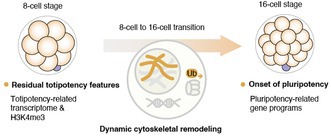

**Abbreviations**
DAPI: 4′,6‐diamidino‐2‐phenylindole
GO: Gene Ontology
PBS: phosphate‐buffered saline
PCA: principal component analysis
PrE: primitive endoderm
TE: trophectoderm

Following fertilization, mammalian embryos undergo a series of precisely coordinated molecular and cellular events that establish the foundation for subsequent development. A pivotal process during this period is the maternal‐to‐zygotic transition, during which maternally stored transcripts are progressively degraded and zygotic genome activation initiates *de novo* transcriptional activity [1]. At the eight‐cell stage, blastomeres undergo compaction and establish apical‐basal polarity, characterized by the accumulation of actomyosin and PAR‐αPKC complexes at the apical cortex [2]. This process not only alters cell morphology and adhesion, but also initiates the spatial asymmetry required for lineage specification [3]. The establishment of polarity enables differential activation of the HIPPO‐YAP signaling pathway, which acts as a key determinant of the first cell‐fate decision [4]. In polarized outer cells, HIPPO signaling is inactive, allowing nuclear localization of YAP and activation of TEAD‐mediated transcription, thereby inducing trophectoderm (TE)‐specific gene expression such as *Cdx2* and *Gata3* [5]. By contrast, inner non‐polarized cells maintain active HIPPO signaling, leading to YAP phosphorylation and cytoplasmic retention, which suppresses TE‐specific programs and promotes inner cell mass identity [5, 6]. This spatially coordinated signaling cascade marks the first lineage segregation, in which outer cells differentiate into the TE and inner cells form the ICM, establishing the foundation for the embryonic and extraembryonic compartments of the blastocyst [7]. Recent studies have further revealed that transcription factors such as NR5A2 and TFAP2C serve as crucial molecular links connecting ZGA (i.e. zygotic genome activation) with the initiation of the first lineage segregation, thereby promoting a bipotent regulatory state at the eight‐cell stage [8, 9]. Nevertheless, the global molecular landscape and regulatory dynamics underlying this transition remain to be fully elucidated, warranting further investigation.

Embryonic totipotency represents a foundational concept in developmental biology, defined as the capacity of a single cell to give rise to all embryonic and extraembryonic lineages, ultimately forming a complete organism [10]. This property underlies the earliest developmental decisions, coordinating the establishment of distinct cell lineages and tissue architectures during embryogenesis [11]. Studies on mammalian totipotency began in the 1950s to 1960s, when researchers first isolated individual blastomeres and cultured them within empty zona pellucida to assess their developmental potential. A landmark finding by Tarkowski in 1959 demonstrated that a single blastomere from a two‐cell stage mouse embryo could develop into a complete adult, providing the first experimental proof of totipotency [12]. Later studies confirmed that mouse embryos maintain totipotent potential up to the four‐cell stage, retaining the ability to contribute to both embryonic and extraembryonic tissues [13, 14]. These pioneering works, employing approaches such as single‐cell culture and lineage tracing, not only verified the totipotent nature of early blastomeres, but also offered early insights into the molecular and cellular mechanisms governing lineage segregation. Despite these advances, a comprehensive understanding of how totipotency transitions into pluripotency remains technically challenging. Although mouse two‐cell and human eight‐cell embryos are widely recognized as key totipotent stages [15], controversy persists over whether later developmental stages retain residual totipotency [16]. Moreover, complete organismal development requires not only intrinsic cellular competence but also appropriate environmental and biochemical contexts [17]. Therefore, isolated blastomere encapsulation or transplantation often fails to fully recapitulate totipotent development beyond these early stages. Chimeric embryo assays using intact embryos have been developed to more accurately assess totipotency [18], but their inherent technical complexity and multiple manipulations may perturb cellular states and introduce confounding factors, thereby complicating the interpretation of developmental potential.

In the present study, we employed a half of the blastomeres biopsy approach, in which half of the blastomeres were removed from embryos and the remaining cells were cultured *in vitro*. By integrating transcriptomic, chromatin immunoprecipitation‐sequencing, immunofluorescence and embryo transfer, we systematically compare the totipotency dynamics in mouse embryos at the two‐cell, four‐cell and eight‐cell stages. Additionally, using low‐input proteomic profiling, we explored the molecular timeline of the transition from totipotency to pluripotency. Our findings demonstrate that eight‐cell stage mouse embryos retain partial totipotent potential and also that ubiquitination‐mediated cytoskeletal degradation emerged as a characteristic molecular event during the transition from late totipotent to pluripotent states. This work not only broadens the understanding of developmental plasticity in mouse embryogenesis, but also offers important clues to the molecular processes underlying the transition from totipotency to pluripotency.

## Materials and methods

### Ethics statement

All animal experiments were conducted in accordance with the guidelines approved by the Institutional Animal Care and Use Committee and the Ethics Committee of the Institute of Animal Science at Tongji University (Approval No. TJAA06424101).

### Animals and collection of normal mouse embryos

Specific pathogen‐free mice were housed in the animal facility of Tongji University (Shanghai, China). To obtain metaphase II (MII) oocytes and staged embryos, female ICR mice (8–10 weeks old) were subjected to induced superovulation via intraperitoneal injection of 5–10 IU pregnant mare serum gonadotropin (San‐sheng Pharmaceutical, Xinxiang, China), followed by 10 IU human chorionic gonadotropin (San‐sheng Pharmaceutical) 48 h later. Cumulus‐oocyte complexes were collected from the ampulla of the oviduct 14–16 h post‐human chorionic gonadotropin administration and co‐incubated for fertilization with capacitated (1 h) cauda epididymal sperm from ICR male mice in human tubal fluid medium (M1135; Nanjing Aibei Biotechnology, Nanjing, China) for 4–6 h. Fertilized zygotes were washed and cultured under mineral oil (M2470; Nanjing Aibei Biotechnology) at 37 °C. Embryos were collected/observed in KSOM medium (M1435; Nanjing Aibei Biotechnology) at 23–43 h (two‐cell), 43–55 h (four‐cell) and 55–67 h (eight‐cell) post‐fertilization. After reaching the 16‐cell stage, embryos were transferred to CZB medium (M1655; Nanjing Aibei Biotechnology) for further culture. Early blastocysts embryos were collected at 67–72 h post‐fertilization, middle blastocyst embryos were collected at 80–84 h post‐fertilization and late blastocyst stage embryos were observed at 100–109 h post‐fertilization.

### Half of the blastomeres biopsy

Briefly, embryos were immobilized with a holding pipette, and the zona pellucida was pierced using a sterilized biopsy needle to aspirate half of the blastomeres, followed by *in vitro* culture. Half of the blastomeres biopsy was performed on a micromanipulation stage maintained at 37 °C. Multiple 10‐μL microdroplets of M2 medium (M1240; Nanjing Aibei Biotechnology) were prepared in a micromanipulation dish, overlaid with mineral oil, and equilibrated at 37 °C with 5% CO_2_ for 15 min. Holding pipettes and biopsy needles were symmetrically mounted on micromanipulator arms and aligned parallel to the stage. Embryos were transferred into pre‐warmed M2 microdroplets, immobilized with a holding pipette and subjected to zona pellucida penetration using a biopsy needle to aspirate half of the blastomeres from each embryo. Biopsied embryos were transferred via mouth pipette to KSOM (potassium‐supplemented simplex optimized medium) medium microdroplets and cultured at 37 °C under 5% CO_2_. Biopsied embryos were further cultured for 2–3 days post‐biopsy until the blastocyst stages for subsequent morphological assessment and sample collection.

### Embryo immunofluorescence

Embryos were washed three times with 1× phosphate‐buffered saline (PBS) and fixed with 4% paraformaldehyde at room temperature for 5 min. After fixation, embryos were rinsed three times in 1× PBS and incubated in blocking solution (10% fetal bovine serum containing 0.2% Triton X‐100) at 4 °C overnight. Primary antibodies were sequentially added and incubated at 4 °C overnight 1 : 50 for GATA2 antibody (11 103‐1‐AP; Proteintech, Rosemont, IL, USA) and 1 : 40 for NANOG antibody (AF2729‐S; R&D Systems, Minneapolis, MN, USA). Following three washes with 1 × PBS, secondary antibodies were co‐incubated with 4′,6‐diamidino‐2‐phenylindole (DAPI) (D9542‐1MG; Sigma‐Aldrich, St Louis, MO, USA; 1 μg·mL^−1^) at room temperature for 2 h in the dark. Stained embryos were washed three times with 1× PBS, transferred to glass‐bottom confocal dishes and imaged using a confocal microscope. Imaging was performed using na LSM‐100 (Zeiss, Oberkochen, Germany) confocal laser scanning microscope.

### Vasectomy and embryo transfer

Healthy 8‐week‐old male ICR mice were anesthetized with tribromoethanol (Avertin; Sigma‐Aldrich; T48402; 200 Μl per 10 g of body weight) and immobilized on thermostatic heating pad at a 37 °C. After disinfecting the surgical area with povidone‐iodine, a 1–1.5 cm midline abdominal incision was made to expose the bilateral vas deferens. Bilateral vas deferens were ligated at two points with 5–0 absorbable sutures and transected between ligations. Postoperatively, mice were maintained on a heating pad at 37 °C until fully recovered and housed individually.

ICR female mice in estrus (with pronounced vulvar swelling) were paired with vasectomized males. Vaginal plug confirmation designated pseudopregnancy day 0.5. Prior to transfer, mice were anesthetized via intraperitoneal tribromoethanol injection. Upon anesthesia induction, bilateral dorsal incisions were made to expose oviducts. A 1‐mL syringe needle was used to puncture the oviductal ampulla and embryos were transferred into the oviduct lumen via a glass capillary connected to a mouth pipette. After wound closure, mice were revived on a heating pad at 37 °C.

### Low‐input proteomics mass spectrometry

A microinjection dish was prepared with a 50 μL drop of acid solution and several 50‐μL drops of PBS, covered with mineral oil, and pre‐warmed in an incubator at 37 °C with 5% CO_2_ for at least 15 min. Before lysis, a heating stage (37 °C) was activated and 50 embryos were transferred into the acid solution using a capillary tube. Once the zona pellucida was completely digested, the embryos were washed three times in PBS. The zona‐free embryos were then transferred into a clean 500‐0μL low‐retention centrifuge tube and stored at −80 °C. After collecting all samples, mass spectrometry was performed using a nano‐liquid chromatography quadrupole orbitrap mass spectrometer, with all mass spectrometry and protein quantification outsourced to the analytical testing center of Shanghai Jiao Tong University (Shanghai, China).

### Data processing and analysis

Transcriptomic data were reanalyzed from previously published datasets [19] and processed using the Seurat R package (version 5.1.0, Rahul Satija, at the New York Genome Center, New York, NY, USA, https://github.com/satijalab/seurat/releases), for clustering and differential expression. Initially, cells with fewer than 5000 detected genes were filtered out, and normalization was performed using the NormalizeData function with default parameters. Three thousand highly variable genes identified by the ‘vst’ method were applied for principal component analysis (PCA) and subsequent clustering. After scaling, dimensionality reduction was first performed using PCA, and UMAP (i.e. uniform manifold approximation and projection) visualization was then generated specifically based on a curated subset of totipotency‐ and pluripotency‐associated genes. A shared nearest neighbor graph was constructed using 30 principal components, and clustering was performed with a resolution of 0.6 using the FindClusters function. Pearson correlation analysis was performed using the cor() function in the R stats package (version 4.4.1, the R Core Team, at the R Foundation for Statistical Computing, Vienna, Austria, https://cran.r‐project.org/), to assess the relationships between the expression levels of genes associated with totipotency, pluripotency and the ubiquitin‐proteasome pathway. Correlation matrices were visualized using the corrplot R package (version 0.92, Taiyun Wei, at the Department of Statistics, Renmin University of China, Beijing, China, https://cran.r‐project.org/package=corrplot).

For low‐input proteomics data, we utilized the ClusterGVis package (version 0.1.1, Zhang jun, at the School of Life Science and Technology, China Pharmaceutical University, Nanjing, Jiangsu Province, China), for gene clustering analysis. ClusterGVis applies the Mfuzz algorithm, a fuzzy clustering approach suitable for analyzing time‐series or condition‐dependent gene expression data. Expression matrices were exported from the Seurat object, and the clusterData function was used to cluster genes based on their expression levels or protein abundance across different cell subtypes. Functional enrichment of each cluster was performed using the enrichCluster function, and results were visualized using visCluster. Gene Ontology (GO) (http://geneontology.org) enrichment analysis of selected gene sets was conducted using the clusterProfiler R package (version 4.12.2, Guangchuang Yu, at the Department of Bioinformatics, School of Basic Medical Sciences, Southern Medical University, Guangzhou, China, https://bioconductor.org/packages/clusterProfiler/), whereas statistical comparisons were performed using the ggsignif package (version 0.6.4, Constantin Ahlmann‐Eltze, at the European Molecular Biology Laboratory, Heidelberg, Germany, and Indrajeet Patil, at the Max Planck Institute for Human Development, Berlin, Germany, https://cran.r‐project.org/package=ggsignif).

Lists of totipotent, pluripotent and maternal genes were obtained from a previous study [20]. H3K4me3 chromatin immunoprecipitation‐sequencing data were also sourced from published datasets [21]. The H3K4me3 signal data were imported from bigWig files, with signal direction corrected according to sample identity. Transcription start site regions (±500 bp) were defined separately for totipotent and pluripotent gene sets. Signal matrices were generated using the normalizeToMatrix function of EnrichedHeatmap R package (version 1.34.0, Zuguang Gu, German Cancer Research Center, Heidelberg, Germany, https://bioconductor.org/packages/EnrichedHeatmap/), with 50‐bp binning, and averaged across biological replicates within each developmental stage group. The resulting matrices were reshaped into long format for visualization. Finally, line plots were created using the ggplot2 R package (version 3.5.1, Hadley Wickham, Posit PBC, Boston, MA, USA, https://cran.r‐project.org/package=ggplot2), with developmental stages displayed in facets and totipotent/pluripotent signals overlaid to enable direct comparison of H3K4me3 enrichment patterns around transcription start site regions.

## Statistical analysis

Data are shown as the mean ± SEM. Statistical differences between two groups were evaluated using non‐parametric Wilcoxon rank‐sum tests unless otherwise specified. All statistical analyses were carried out using prism (GraphPad Software Inc., San Diego, CA, USA) or r (R Foundation, Vienna, Austria). *P* < 0.05 was considered statistically significant.

## Results

### Multi‐omics revealed dynamic shifts from totipotency to pluripotency during early embryonic development

**Fig. 1:**
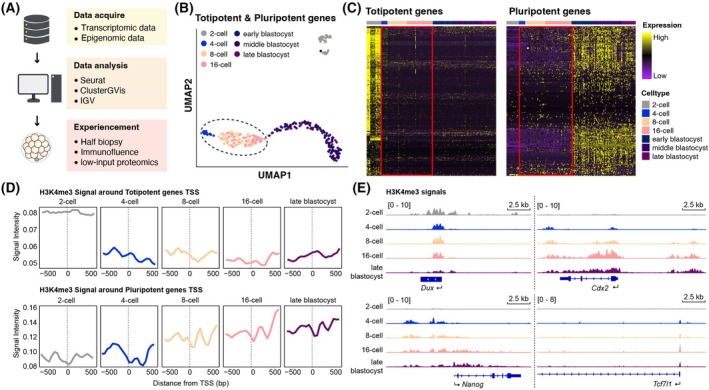
Multi‐omics analysis reveals dynamic shifts in totipotency and pluripotency during early embryogenesis. (A) Schematics of the whole research. (B) PCA of single‐cell transcriptomes from zygote to blastocyst stages by using totipotent and pluripotent genes. Embryos from two‐cell to 16‐cell stages cluster separately from early to late blastocyst stages, reflecting transcriptional divergence between totipotent and pluripotent states. (C) Hierarchical clustering of totipotent and pluripotent genes expression. (D) Profiles of the H3K4me3 signal density around promoters of totipotent and pluripotent genes. H3K4me3 signal density was calculated using H3K4me3 signals with 50‐bp resolution. (E) Representative genome browser snapshots show H3K4me3 enrichment at *Dux* (totipotency), *Cdx2* (trophectoderm), *Nanog* (epiblast) and *Tcf7l1* (primitive endoderm) across different stages.

Leveraging published single‐cell transcriptomic datasets, we performed hierarchical clustering analysis to systematically dissect the temporal expression dynamics of core regulatory genes governing totipotency and pluripotency during embryogenesis (Fig. 1A). UMAP analysis based on totipotent and pluripotent gene expression clustered the cells, revealing that transcriptional profiles from four‐cell to 16‐cell stage embryos were highly similar and grouped into a distinct cluster. By contrast, samples from the early blastocyst, mid blastocyst and late blastocyst stages formed a separate cluster (Fig. 1B). Further hierarchical clustering analysis demonstrated that two‐cell stage embryos were characterized by enriched expression of totipotent genes, whereas the early blastocyst to late blastocyst stages specifically activated pluripotent gene networks (Fig. 1C). Notably, four‐cell, eight‐cell and 16‐cell stage embryos displayed analogous transcriptional patterns, marked by co‐expression of partial totipotent and pluripotent regulators and dynamic transitional features, suggesting this phase represents a critical window for the progressive loss of cellular fate plasticity (Fig. 1C).

To further investigate epigenomic alterations in developmental potency, we examined the dynamic patterns of H3K4me3 modifications, which are active histone marks associated with transcriptional initiation and gene activation, at the promoter regions of totipotent and pluripotent genes across different developmental stages. Notably, promoters of totipotent genes exhibited the strongest H3K4me3 signal at the two‐cell stage, which sharply declined beginning at the four‐cell stage and remained consistently low throughout subsequent stages, including the four‐cell, eight‐cell, 16‐cell and late blastocyst (Fig. 1D). By contrast, promoters of pluripotent gene displayed an opposing trend, with minimal H3K4me3 enrichment at the two‐cell stage followed by a gradual increase as development progressed from the four‐cell stage to the blastocyst (Fig. 1D). Additionally, we further investigated the H3K4me3 modification profiles at key lineage‐specific gene loci to gain deeper insights into epigenetic regulation during early embryogenesis. For totipotency gene *DUX*, H3K4me3 modifications were significantly enriched in two‐cell embryos, with comparable enrichment levels maintained from the four‐cell to 16‐cell stages, followed by a sharp decline at the blastocyst stage (Fig. 1E). By contrast, the trophoblast (TE) lineage gene *Cdx2* exhibited negligible H3K4me3 enrichment at the two‐cell stage, which progressively increased from the four‐cell stage onward and reached a significant elevation by the 16‐cell stage (Fig. 1E). Similarly, the epiblast lineage gene *Nanog* lacked H3K4me3 marks at the two‐cell stage but showed gradual enrichment starting at the four‐cell stage, peaking at the blastocyst stage, indicative of temporally regulated pluripotency activation (Fig. 1E). Furthermore, the primitive endoderm (PrE) lineage gene *Tcf7l1* displayed no detectable H3K4me3 enrichment at the two‐cell stage but maintained stable modification levels across subsequent stages (four‐cell stage to blastocyst) (Fig. 1E).

Our observations are consistent with the possibility that embryos at the four‐cell to eight‐cell stages maintain partial totipotent characteristics, whereas 16‐cell embryos display an early bias toward TE lineage differentiation. Accordingly, systematic investigation of developmental potential during cleavage‐stage progression could provide valuable clues regarding the dynamics of early embryonic plasticity.

### Half of the blastomeres biopsy reveals totipotent‐like potential in eight‐cell stage embryos

**Fig. 2:**
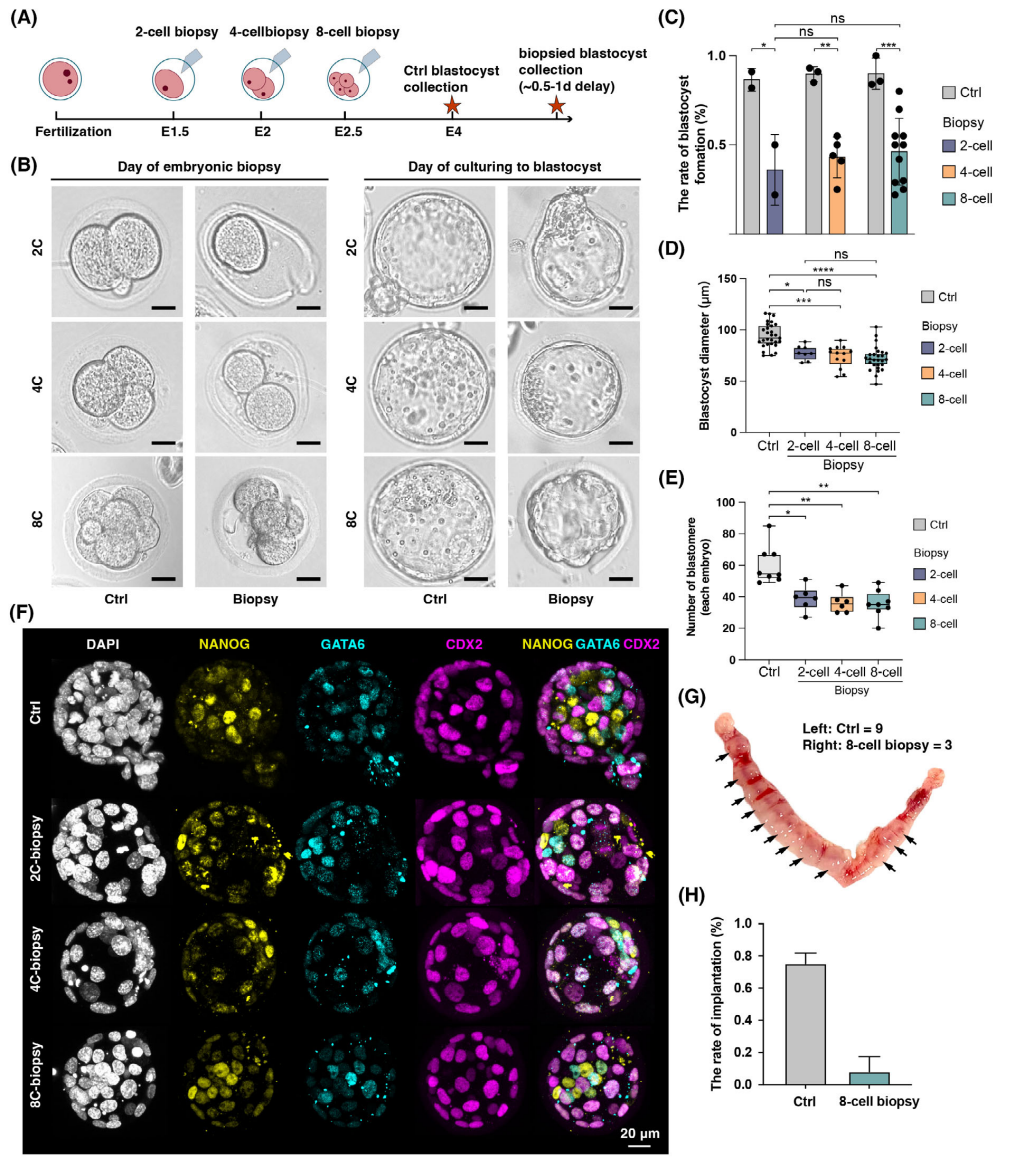
Developmental potential of biopsied embryos reveals residual totipotency in the eight‐cell stage. (A) Schematic timeline showing embryo biopsy at the two‐cell, four‐cell and eight‐cell stages, followed by collection of control and biopsied blastocysts. Biopsied blastocysts were collected with a 0.5–1‐day delay relative to controls. (B) Representative images for blastocyst morphology of control and biopsied embryos at day 0 (left) and days 2–3 (right). The corresponding quantification and statistical analyses are provided in Fig. 2C. Scale bar = 20 μm. (C) Blastocyst formation rates of control and biopsied embryos. Each dot represents one independent experiment, with 25–30 embryos analyzed per group in each experiment. Bars indicate the mean ± SEM; non‐parametric Wilcoxon rank sum tests. (D) Blastocyst diameter quantification of controls and biopsied embryos. Data were obtained from three independent experiment. Data are the mean ± SEM; ns, not significant, *P* > 0.05, *P < 0.05, **P < 0.01 and ***P < 0.001; non‐parametric Wilcoxon rank‐sum tests. (E) Quantification of blastomere number in control and biopsied blastocysts. Data were obtained from three independent experiment. Data are the mean ± SEM; ns, not significant, *P* > 0.05, *P < 0.05, **P < 0.01 and ***P < 0.001; non‐parametric Wilcoxon rank‐sum tests. (F) Immunofluorescence of control and biopsied blastocysts. Data were obtained from three independent experiment. DAPI (white), NANOG (yellow), GATA6 (cyan) and CDX2 (purple). Scale bar = 20 μm. (G) Representative images showing *in vivo* implantation sites of control (left) and eight‐cell biopsy (right) groups at embryonic day 7.5 after uterine dissection. Arrows indicate implanted embryos. (H) Quantification of implantation rates for control and eight‐cell biopsy embryos. Data were obtained from three independent embryo transfer experiments, with 15 embryos transferred per group in each experiment. Error bars represent the mean ± SEM across biological replicates.

To investigate developmental plasticity, we performed half of the blastomeres removal in two‐cell, four‐cell and eight‐cell stage embryos using embryo biopsy techniques (Fig. 2A). This method enabled reliable isolation of blastomeres at distinct developmental stages, establishing a robust methodological framework for downstream analyses (Fig. 2A).

Blastocyst formation following biopsy of two‐cell embryos was monitored during subsequent culture, providing a reference baseline for comparisons with later developmental stages. To rule out potential procedural artifacts, unmanipulated embryos cultured in parallel served as experimental controls. Our results demonstrated that embryos in the control group exhibited normal developmental progression with low fragmentation rates, confirming that biopsy was the sole experimental variable (Fig. 2B). In four‐cell embryos, blastocyst formation rates following the removal of half the blastomeres were comparable to those of the two‐cell group, with no significant morphological abnormalities, indicating preserved developmental potential at this stage (Fig. 2B). Notably, although eight‐cell embryos subjected to biopsy displayed TE cells with uneven borders rather than the smooth polygonal outlines seen in controls, approximately half of the eight‐cell embryos subjected to biopsy were still able to develop into blastocyst‐like structures, indicating that embryos at this stage preserve a degree of totipotent‐like potential (Fig. 2B). To systematically evaluate the developmental potential of embryos across different stages, we integrated multi‐batch experimental data and performed statistical analyses to compare blastocyst formation rates among different groups. Our results showed that, although blastocyst formation rates were significantly reduced in embryos biopsied at the two‐cell, four‐cell and eight‐cell stages compared to unmanipulated controls, no significant differences were detected among the biopsied groups themselves, indicating a comparable impact of blastomere removal across these stages (Fig. 2C).

To assess the impact of biopsy on blastocyst development, we measured blastocyst diameters in experimental (two‐cell, four‐cell and eight‐cell biopsy groups) and control groups (Table S1). Quantitative data showed control blastocyst diameters of 94.46 ± 11.65 μm, whereas biopsied groups ranged from 72.02 ± 11.50 μm to 77.70 ± 6.58 μm (Fig. 2D). After an additional 0.5–1 day of culture to allow biopsied embryos to reach comparable developmental stages, all biopsied groups exhibited significantly smaller blastocyst diameters compared to controls (*P* < 0.001), although no significant differences were observed among the biopsy groups themselves (Fig. 2D). Quantification of total cell numbers by DAPI nuclear staining further revealed that blastocysts derived from biopsied embryos contained significantly fewer cells than controls (Fig. 2E). To investigate biopsy‐induced effects on lineage specification, we performed multiplex immunofluorescence staining for lineage‐specific markers in experimental blastocysts, including NANOG (epiblast, EPI), GATA6 (primitive endoderm, PrE) and CDX2 (trophectoderm, TE). Immunofluorescence analysis showed that all biopsied blastocysts exhibited spatially segregated expression of these lineage markers, comparable to unmanipulated controls (Fig. 2F). These results indicate that blastocysts derived from biopsied embryos at the two‐cell, four‐cell and eight‐cell stages not only retained normal morphology, but also successfully established molecularly defined embryonic lineages.

To further investigate whether the totipotent capacity of biopsied eight‐cell stage embryos support embryo implantation and later intrauterine development, 15 early blastocysts from the control and eight‐cell biopsied group were transferred into contralateral oviducts of recipient mice separately. Dissection at embryonic day 7.5 (5 days post‐transfer) revealed successful implantation in the eight‐cell biopsy group (Fig. 2G). Although implantation rates were lower compared to controls, a subset of embryos from the eight‐cell biopsy group successfully implanted (Fig. 2H), demonstrating that blastomeres at this stage preserve residual developmental potential and represent a transitional state near the end of the totipotent window.

### Low‐input proteomics revealed ubiquitin‐proteasome pathway involvement in the totipotency‐to‐pluripotency transition

**Fig. 3:**
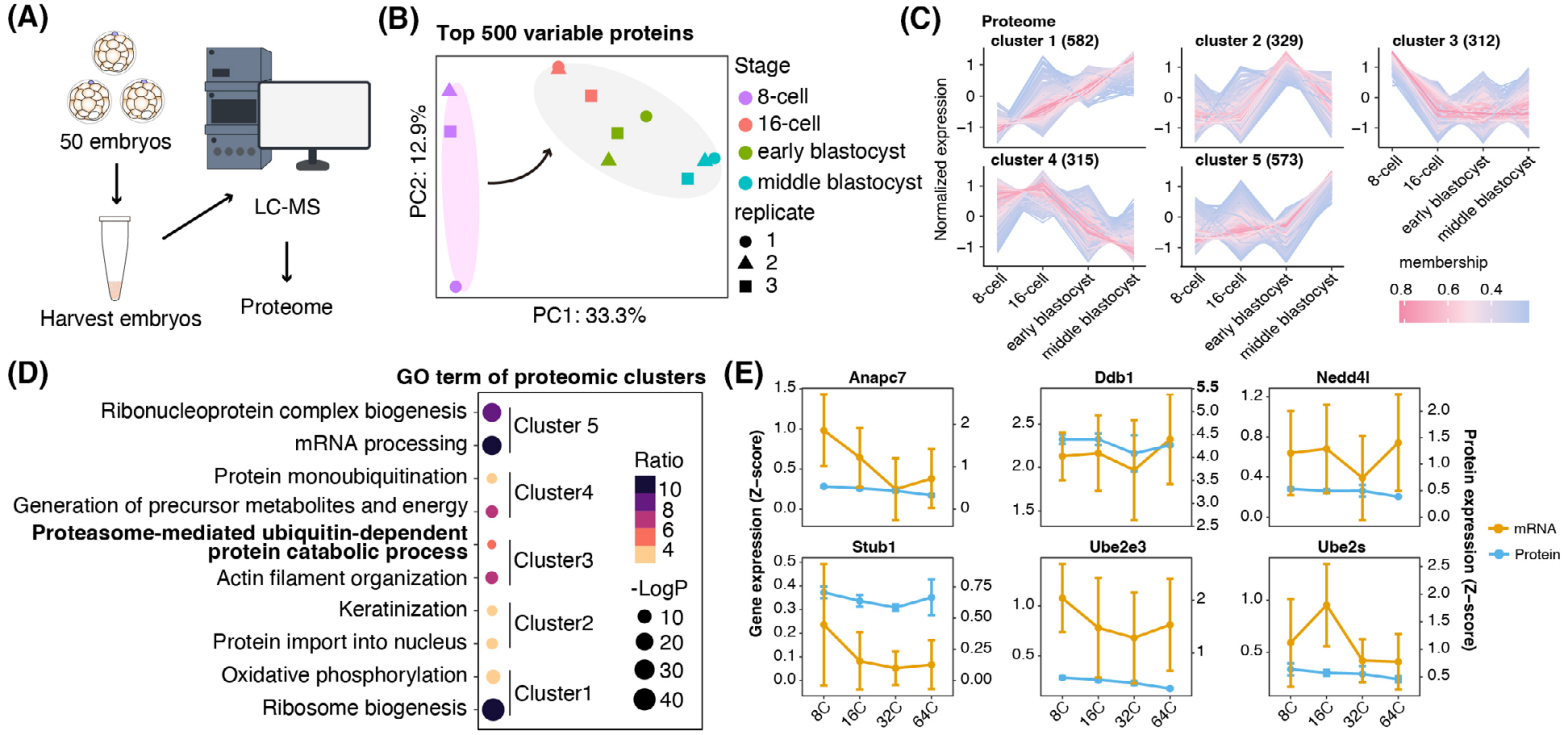
Proteomic profiling reveals that factors involving in ubiquitination‐proteolysis pathway were enriched at the eight‐cell stage. (A) Experimental workflow of low‐input proteomics mass spectrometry. (B) PCA scatter plot based on the top 500 most variable proteins across embryos at different stages (*n* = 3). (C) Line plot showing protein level changes across different embryonic stages (from eight‐cell to middle blastocyst stage). The *x*‐axis represents different developmental stages, whereas the *y* ‐axis represents normalized intensity ratios in each stage. (D) Dot plot of protein functional clustering across different five clusters. (E) Line plot illustrates the gene and protein expression levels of molecules involved in the proteasome‐mediated ubiquitin‐dependent protein degradation pathway across different stage. Error bars represent the mean ± SEM across biological replicates.

Proteins directly participate in and determine the physiological activities of cells. To further detect the molecular characteristics during the final transition from totipotency to pluripotency from the eight‐cell stage, we employed low‐input proteomics to systematically profile dynamics of protein during fate transitions from the eight‐cell stage to the middle blastocyst stage (Fig. 3A). Using low‐input proteomic profiling, we analyzed 12 biological replicates (threre replicates per stage, each containing 50 embryos) across four developmental stages (eight‐, 16‐cell stages, early blastocyst and middle blastocyst stages). PCA of the top 500 highly variable proteins revealed distinct clustering by developmental stage (Fig. 3B). Notably, eight‐cell embryos formed an isolated cluster, whereas 16‐cell, early and middle blastocyst embryos grouped closely together. This distribution highlights the eight‐cell to 16‐cell transition as a pivotal stage of proteomic remodeling, establishing the eight‐cell stage as the most distinct from later developmental stages (Fig. 3B).

Based on proteomic data across embryonic developmental stages, we identified five temporally regulated protein clusters through hierarchical clustering analysis (Fig. 3C). Cluster 1 (582 proteins) showed sustained upregulation from the eight‐cell to middle blastocyst stage, cluster 2 (329 proteins) exhibited stage‐specific upregulation at the early blastocyst stage, cluster 3 (312 proteins) peaked at the eight‐cell stage and declined sharply from the 16‐cell stage onward, cluster 4 (315 proteins) maintained high abundance from the eight‐cell to 16‐cell stages before gradually decreasing after the early blastocyst stage, and cluster 5 (573 proteins) was specifically enriched at the middle blastocyst stage (Fig. 3C and Table S2). GO clustering analysis revealed distinct functional patterns, with cluster 1 associated with oxidative phosphorylation and ribosome biogenesis, cluster 2 with keratinization and regulation of nuclear protein transport, cluster 3 with ubiquitination‐mediated proteolysis, endoplasmic reticulum stress response and actin polymerization, cluster 4 with polyubiquitination modification and energy precursor metabolism, and cluster 5 with ribosome assembly and mRNA synthesis pathways (Fig. 3D).

**Fig. 4:**
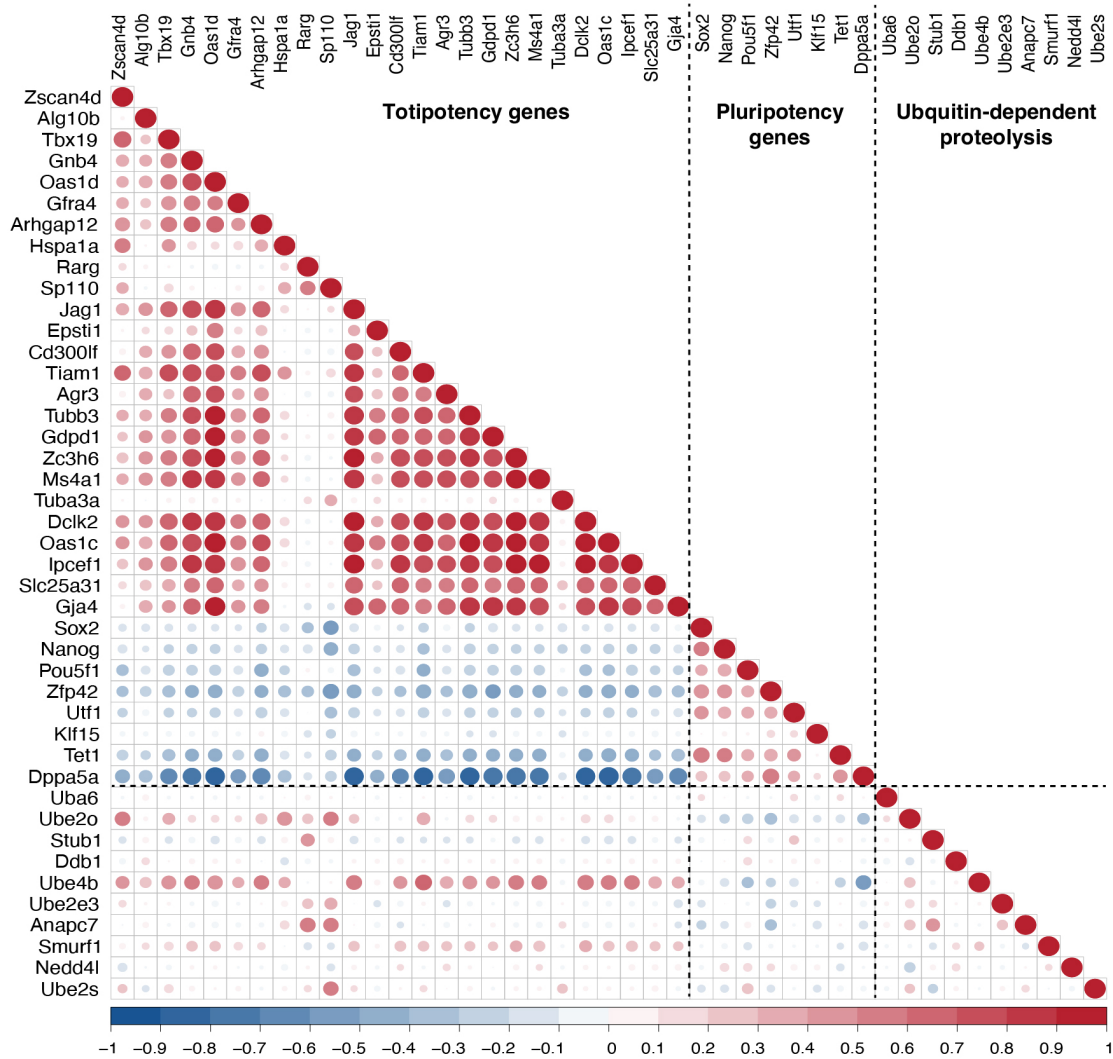
Gene expression correlations in totipotency, pluripotency and ubiquitin‐dependent proteolysis. Correlation analysis based on single‐cell transcriptomic data revealed that the expression of totipotent gene clusters showed positive correlations with ubiquitin‐dependent proteolysis genes, whereas the expression of core pluripotent genes was negatively correlated with ubiquitin‐dependent proteolysis genes.

To further investigate the dynamic changes in the ubiquitin–proteasome pathway, we integrated transcriptomic and proteomic data to examine the expression and abundance of representative components of this pathway, including *ANAPC7*, *DDB1*, *NEDD4L*, *STUB1*, *UBE2E3* and *UBE2S*, across different developmental stages. Although these genes were not directly enriched in cluster 3, the GO analysis of this cluster revealed a strong association with ubiquitination‐related processes, prompting us to explore additional core members of the pathway. We found that the trends in both protein abundance and gene expression for ANAPC7, DDB1, NEDD4L and STUB1 were largely consistent, further validating our proteomics results. To explore the relationship between the ubiquitin‐proteasome pathway and potency, we performed correlation analysis on the expression of key molecular associated with totipotency, pluripotency and ubiquitin‐proteasome pathway. Specifically, the expression of totipotent genes (*Zscan4d*, *Tbx19* and *Dppa4a*) showed significant positive correlations with genes involved in ubiquitin‐mediated proteolysis, whereas genes from the core pluripotency regulatory network (*Nanog*, *Sox2* and *Pou5f1*) displayed negative correlations with ubiquitin‐mediated proteolysis (Fig. 4A). Collectively, these findings reveal a significant enrichment of ubiquitin‐proteasome‐related molecules in eight‐cell embryos, implicating their potential role in the totipotency‐to‐pluripotency transition.

### Cytoskeletal and totipotency‐associated maternal proteins accumulate upon proteasome inhibition

**Fig. 5:**
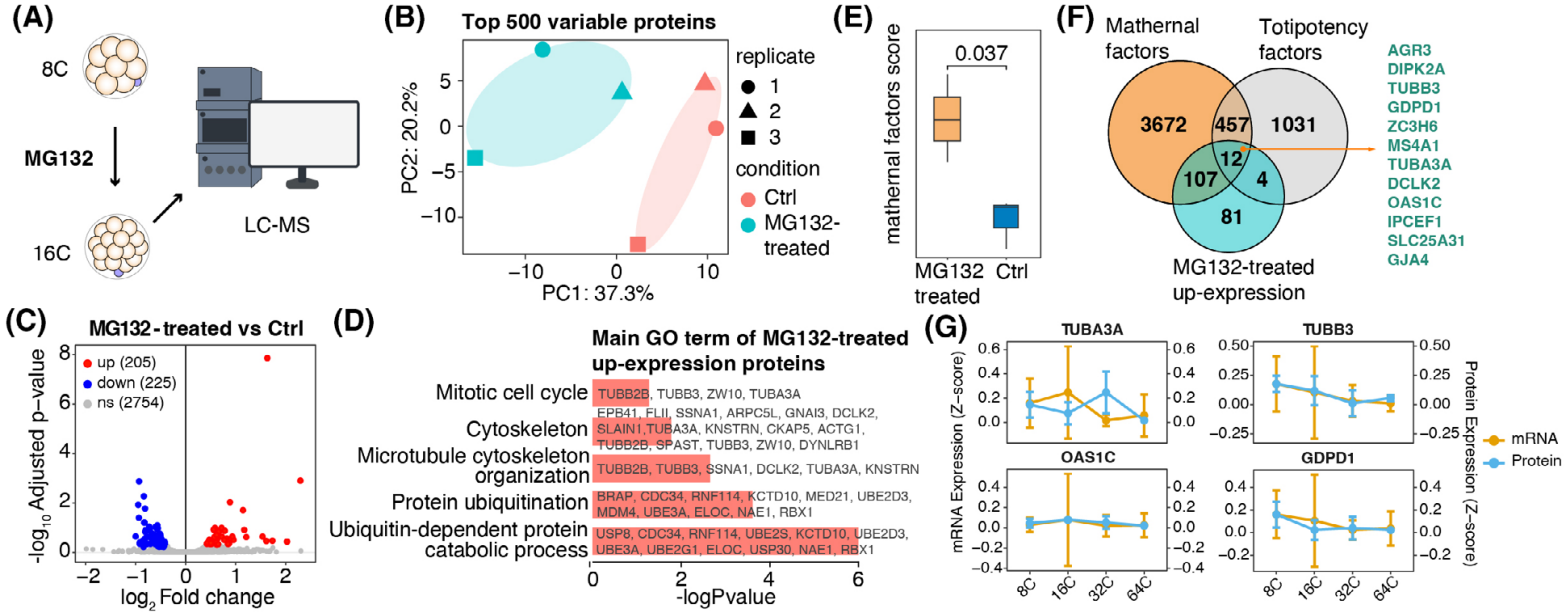
Cytoskeletal proteins as major targeted of ubiquitin‐dependent proteolysis degradation. (A) Experimental workflow of low‐input proteomics following MG132 treatment. (B) PCA showing the divergence of MG132‐treated embryos from control embryos (*n* = 3). (C) Volcano plot displaying 89 upregulated proteins (red) and 114 downregulated proteins (blue) after MG132 treatment (absolute log_2_ fold change > 1, adjusted *P* < 0.05). (D) GO analysis of the upregulated proteins in the MG132‐treated group. (E) Boxplot comparing maternal protein abundance between MG132‐treated and control groups (*n* = 3). Data are presented as the mean ± SEM; **P < 0.05; non‐parametric Wilcoxon rank‐sum tests. (F) Venn diagram showing the overlap of MG132‐upregulated proteins with totipotent and maternal factors. (G) Line plot showing the gene and protein expression levels of the molecules involved in the overlapping genes from Fig. 5F (*n* = 3).

To identify key proteins degraded via the ubiquitin‐dependent pathway, we treated eight‐cell stage embryos with the proteasome inhibitor MG132 (10 μm) and analyzed proteomic changes at the 16‐cell stage (Fig. 5A). We collected 16‐cell embryos from untreated controls and MG132‐treated groups, with three biological replicates per group. PCA analysis showed a partial separation between the two groups, with MG132‐treated embryos predominantly distributed on the left side and controls clustering on the right, indicating a general trend of proteomic remodeling following MG132 treatment (Fig. 5B). Differential analysis identified 204 proteins with significantly increased abundance and 225 proteins with decreased abundance in the MG132 group (Fig. 5C). Functional annotation of the upregulated proteins highlighted that, beyond the global remodeling of ubiquitination‐related factors, changes in cytoskeletal proteins were particularly pronounced (Fig. 5D).

Given the critical role of maternal factors in the establishment and maintenance of totipotency [22, 23], we further examined changes in maternal protein abundance following MG132 treatment. We found that MG132 treatment significantly increased the levels of maternal proteins compared to the control group (Fig. 5E). Notably, among the 204 proteins that were significantly upregulated after MG132 treatment, 12 were identified as both maternal and totipotency‐associated factors (Fig. 5F and Table S3). To further validate these findings, we focused on the subset of these 12 proteins that were detected in our proteomic data of normal embryos, including TUBB3, TUBA3A, OAS1C and GDPD1 (Fig. 5G). We observed that, across the stages analyzed from the eight‐cell to the blastocyst stages, both *TUBB3* and *GDPD1* displayed their highest expression levels at the eight‐cell stage in both the transcriptomic and proteomic datasets (Fig. 5G).

## Discussion

Early mammalian development proceeds through a progressive restriction of cellular potency, during which blastomeres gradually lose totipotency and acquire pluripotent or lineage‐committed states. In the present study, we re‐evaluated the developmental potential of eight‐cell stage blastomeres and explored the molecular pathways that may mediate the transition from totipotency to pluripotency. Through blastomere biopsy and half of the blastomeres extraction experiments, we found that embryos at the two‐cell, four‐cell and eight‐cell stages were still able to form blastocysts after removal of half of the blastomeres, with comparable rates of blastocyst formation (approximately 50%) among these groups. Although the implantation efficiency of biopsy‐treated eight‐cell embryos was substantially lower (approximately 15%), the observation of successful implantation events indicates that blastomeres at this stage retain partial developmental plasticity. These findings are consistent with earlier reports showing that embryos can continue development after blastomere biopsy, albeit often with delayed progression or reduced success rates [24, 25]. Taken together, our data support the notion that eight‐cell blastomeres represent a transitional state characterized by declining but not entirely lost totipotent potential. To further identify molecular mechanisms associated with this transition, we performed low‐input proteomic profiling of embryos from the eight‐cell to blastocyst stages. Enrichment analysis revealed that the ubiquitin‐proteasome pathway was specifically active at the eight‐cell stage and declined thereafter, suggesting its involvement in remodeling the cellular architecture accompanying the loss of totipotency. Inhibition of the proteasome with MG132 resulted in accumulation of cytoskeletal proteins and impaired degradation of maternal components, consistent with the hypothesis that timely ubiquitin‐mediated turnover of maternal proteins is essential for developmental progression. Quantitative comparison of protein abundance between the eight‐cell and 16‐cell stages identified multiple cytoskeletal and cytoskeleton‐associated proteins exhibiting significant downregulation. These data suggest that cytoskeletal remodeling, regulated by ubiquitin‐dependent proteolysis, may be an important molecular process contributing to the totipotency‐to‐pluripotency transition.

Previous studies have reported that the ubiquitin‐proteasome system becomes activated during the maternal‐to‐zygotic transition, facilitating the clearance of maternal proteins and establishing a proteomic landscape permissive for zygotic genome activation [26, 27, 28, 29]. Dysregulation of this system has been shown to delay the onset of ZGA and impair embryonic viability, underscoring its pivotal role in developmental reprogramming [27]. Our proteomic data extend this understanding by linking ubiquitin‐dependent proteolysis to cytoskeletal remodeling, which represents a major structural reorganization event accompanying the transition from the eight‐cell to the morula stage. Several studies in other cellular contexts have demonstrated that the ubiquitin‐proteasome pathway regulates cytoskeletal dynamics through the selective degradation of polarity regulators and actin‐polymerizing factors, such as PAR proteins and vasodilator‐stimulated phosphoprotein, thereby influencing cell shape, adhesion and polarity establishment [30]. Moreover, the crosstalk between the proteasome and microtubule networks has been recognized as a conserved mechanism coordinating cytoskeletal organization and protein localization [31]. Integrating these findings with our results, we speculate that ubiquitin‐dependent degradation of cytoskeletal components may facilitate dynamic changes in cell polarity, compaction and intracellular tension, thereby establishing the mechanical framework required for lineage segregation and blastocyst morphogenesis. Importantly, we also identified TUBB3 as a maternal factor and totipotency‐associated protein that shows marked downregulation during the transition from the eight‐cell to 16‐cell stage, suggesting that the reduction of TUBB3 is closely associated with the exit from totipotency and the establishment of pluripotency. Future mechanistic investigations, such as proteasome inhibition combined with cytoskeletal imaging or polarity marker analysis, will be essential to elucidate how ubiquitin‐mediated proteolysis integrates with morphogenetic remodeling during preimplantation embryo development.

In summary, our findings establish the link between dynamic regulation of the ubiquitin‐proteasome pathway and the transition of embryonic potency. We confirm that blastomeres in mouse eight‐cell blastomeres in the mouse embryo maintain residual totipotency and undergo coordinated remodeling of the cytoskeleton mediated by the ubiquitin‐proteasome pathway. Through low‐input proteomics, we demonstrate that the ubiquitin‐proteasome pathway drives this transition by dynamically regulating cytoskeletal remodeling. Although our proteomic data support this model, further quantitative validation of maternal protein dynamics and ubiquitin‐mediated targets will be required to fully delineate their mechanistic contributions. Collectively, these findings provide new insights into how proteolytic regulation contributes to developmental potency transitions and may inform strategies for improving reprogramming efficiency in regenerative medicine.

## Conflicts of interest

The authors declare that they have no conflicts of interest.

## Author contributions

WL conceived and designed the analyses and wrote the paper. XX and LQ conceived the data analysis. CL conceived the low‐input proteomics mass spectrometry. SS conceived and performed the *in vitro* embryo transfer. JQ assisted with experimental work. ZX, JX and HL designed the analyses and supervised the project.

## Supporting information


**Table S1.** List of ctrl and biopsied embryos diameters.

**Table S2.** List of proteins in each cluster.

**Table S3.** List of genes associated with totipotency, pluripotency, maternal origin and differential protein expression upon MG132 treatment.

## Data Availability

Processed data of single cell transcriptomics were downloaded from NCBI GEO database under accession number GSE45719. Processed data of H3K4me3 were downloaded from NCBI GEO database under accession number GSE73952.
